# Angioedema Without Wheals: Challenges in Laboratorial Diagnosis

**DOI:** 10.3389/fimmu.2021.785736

**Published:** 2021-12-08

**Authors:** Anete S. Grumach, Camila L. Veronez, Dorottya Csuka, Henriette Farkas

**Affiliations:** ^1^Clinical Immunology, Faculdade de Medicina, Centro Universitario Faculdade de Medicina ABC (FMABC), Santo Andre, Brazil; ^2^Division of Rheumatology, Allergy and Immunology, Department of Medicine, University of California San Diego, San Diego, CA, United States; ^3^Research Service, San Diego Veterans Affairs Healthcare, San Diego, CA, United States; ^4^Hungarian Angioedema Center of Reference and Excellence, Department of Internal Medicine and Haematology, Semmelweis University, Budapest, Hungary

**Keywords:** hereditary angioedema (HAE), angioedema without wheals, C1 inhibitor (C1-INH), C4, biomarker, diagnosis

## Abstract

Angioedema is a prevailing symptom in different diseases, frequently occurring in the presence of urticaria. Recurrent angioedema without urticaria (AE) can be hereditary (HAE) and acquired (AAE), and several subtypes can be distinguished, although clinical presentation is quite similar in some of them. They present with subcutaneous and mucosal swellings, affecting extremities, face, genitals, bowels, and upper airways. AE is commonly misdiagnosed due to restricted access and availability of appropriate laboratorial tests. HAE with C1 inhibitor defect is associated with quantitative and/or functional deficiency. Although bradykinin-mediated disease results mainly from disturbance in the kallikrein–kinin system, traditionally complement evaluation has been used for diagnosis. Diagnosis is established by nephelometry, turbidimetry, or radial immunodiffusion for quantitative measurement of C1 inhibitor, and chromogenic assay or ELISA has been used for functional C1-INH analysis. Wrong handling of the samples can lead to misdiagnosis and, consequently, mistaken inappropriate approaches. Dried blood spot (DBS) tests have been used for decades in newborn screening for certain metabolic diseases, and there has been growing interest in their use for other congenital conditions. Recently, DBS is now proposed as an efficient tool to diagnose HAE with C1 inhibitor deficiency, and its use would improve the access to outbound areas and family members. Regarding HAE with normal C1 inhibitor, complement assays’ results are normal and the genetic sequencing of target genes, such as exon 9 of *F12* and *PLG*, is the only available method. New methods to measure cleaved high-molecular-weight kininogen and activated plasma kallikrein have emerged as potential biochemical tests to identify bradykinin-mediated angioedema. Validated biomarkers of kallikrein–kinin system activation could be helpful in differentiating mechanisms of angioedema. Our aim is to focus on the capability to differentiate histaminergic AE from bradykinin-mediated AE. In addition, we will describe the challenges developing specific tests like direct bradykinin measurements. The need for quality tests to improve the diagnosis is well represented by the variability of results in functional assays.

## Introduction

Angioedema is a prevailing symptom in different diseases, frequently occurring in the presence of urticaria (1). Recurrent angioedema without urticaria (AE) is considered as a distinct pathology with hereditary (HAE) and acquired (AAE) causes, and several subtypes can be distinguished, although clinical presentation is quite similar in most of them (2). Although the first descriptions of HAE appeared as early as in the XVIII century (3), the first cause of the disease was only identified in 1963 (4) as the deficiency of the inhibitor of C1 esterase (C1-INH) in plasma (HAE-C1-INH) (OMIM #106100), initiating a new era of the complement biochemical analysis in HAE patients, establishing low C1-INH and low C4 plasma levels as biomarkers of HAE-C1-INH (5).

HAE with normal C1-INH (HAE-nlC1-INH) (OMIM #610618) was recognized as a distinct HAE type in 2000 by exclusively affecting female patients and by a relationship between severe outcomes and estrogen (6, 7). Six years later, specific mutations in factor XII gene (*F12*) emerged as the first biomarkers for a new subtype of HAE-nlC1-INH, the HAE-F12, caused by mutations affecting a highly glycosylated region of factor XII encoded by the exon 9 of *F12* (8–10). Regarding the main molecular mechanism leading to HAE-C1-INH and HAE-F12, both culminate in an increased production of the vasoactive peptide bradykinin due to the lack of the kallikrein–kinin system inhibition by C1-INH (11) or due to a facilitated activation of mutated factor XII (10, 12), respectively.

Although a causative mutation cannot be found in a considerable number of patients with HAE-nlC1-INH, new variants have been recently described in new genes and associated as disease causing, such as the change p.Ala119Ser in angiopoietin 1 gene (*ANGPT1*) (13), p.Lys330Glu in plasminogen (*PLG*) (14), p.Met379Lys in kininogen (*KNG1*) (15), p.Arg217Ser in myoferlin (*MYOF*) (16), and p.Thr144Ser in heparan sulfate 3-O-sulfotransferase 6 gene (*HS3ST6*) (Bork et al, 2021) (17). The new mutations not only imply novel mechanisms and systems involved in the pathogenesis of HAE, but also open possibility for new biomarkers and treatment targets.

Idiopathic histaminergic acquired angioedema (AAE-IH) is the most common subtype of AE; the patients are responsive to antihistamines and the etiology is usually unknown (18, 19). These patients probably do not share the main involvement of bradykinin, as well as a smaller group of patients with AAE idiopathic non-antihistaminergic (AAE-InH) (20). An ultra-rare group of patients presents with acquired C1-INH deficiency (AAE-C1-INH) (21, 22). Another rare form of AAE is the angioedema exclusively induced by angiotensin-converting enzyme (ACE) inhibitors (AAE-ACEi), which affects less than 1% of patients taking this class of drug (23).

## Subtypes of Angioedema From a Biochemical Point of View

To differentiate between the many known subtypes of angioedema, specific complement tests need to be performed, which can distinguish between angioedema with or without C1-inhibitor deficiency.

### Hereditary Angioedema due to C1-Inhibitor Deficiency

The diagnosis of the most studied AE subtype, the HAE-C1-INH, can be established most precisely in case the following tests are performed: C1-INH function measurement, the antigenic level of the C1-INH protein, C4 and C1q concentration, as well as titers of anti-C1-INH antibodies (**Table 1**) (24).

**Table 1:** The complement laboratory diagnosis of angioedema subtypes.

Type of HAE	C1-INH function	C1-INH concentration	C4	C1q	Anti-C1-INH antibody	Total function of the classical pathway	Mutation in the *SERPING1*gene	Mutation in genes other than *SERPING1*
HAE-C1-INH Type I	Low	Low	Low	Normal	No	Low	Yes	No
HAE-C1-INH Type II	Low	Normal/Elevated	Low	Normal	No	Low	Yes	No
AAE-C1-INH	Low	Low	Low	Low	Yes*	Low	No	No
AAE-ACEI	Normal	Normal	Normal	Normal	No	Normal	No	No
AAE-InH	Normal	Normal	Normal	Normal	No	Normal	No	No
HAE-nlC1-INH	Normal	Normal	Normal	Normal	No	Normal	No	Yes (*F12, PLG, ANGPT1, KNG1, MYOF*, and *HS3ST6*)
U-HAE	Normal	Normal	Normal	Normal	No	Normal	No	Unknown genetic background

*Anti-C1-INH antibody is not present in all the patients. HAE-C1-INH: Hereditary Angioedema with C1 inhibitor deficiency; AAE-C1-INH: Acquired Angioedema with C1 inhibitor deficiency; AAE-ACEi: Acquired angioedema induced by angiotensin-converting enzyme inhibitors; HAE-nlC1-INH: Hereditary Angioedema with normal C1 inhibitor; U-HAE: Hereditary Angioedema with normal C1 inhibitor and unknown mutation.

Parallel measurement of the function and the antigenic C1-INH level are mandatory to differentiate between HAE-C1-INH type I and type II. In HAE type I, C1-INH concentration and function are low, usually less than 50% of the normal level, as no protein is secreted from the mutated allele (24). On the other hand, HAE-C1-INH type II is characterized by normal or even elevated C1-INH serum levels, along with decreased function of C1-INH, due to the presence of detectable, but non-functional mutant C1-INH (25).

In both types of HAE-C1-INH, the early steps of the complement classical pathway are underregulated, which leads to a more pronounced complement activation (as shown by reduced total classical complement function) and consumption of early complement components (as indicated by low C4 levels). Remarkably, C1-INH measurement is the gold standard for the diagnosis of angioedema subtypes (1, 2), as C4 concentration is often low in systemic autoimmune diseases, such as systemic lupus erythematosus (26).

### Angioedema with Acquired C1-Inhibitor Deficiency

Acquired angioedema with C1-inhibitor deficiency (AAE-C1-INH) is also characterized by decreased C1-INH and C4 levels, as well as by altered C1-INH function, but it is accompanied by decreased levels of C1q and, in a large percent of cases, the presence of anti-C1-INH antibodies. However, there are cases in which C1q levels are normal (21, 27–29). AAE-C1-INH is more rare, with an incidence of 1 for 8.8 patients with HAE-C1INH (21). When further classifying this disease, two types were distinguished. In AAE-C1-INH type I, it was reported that a monoclonal component either of unknown significance or due to a myeloma that had C1-inhibitor-binding ability (generated by lymphoproliferative onco-hematologic or immunoregulatory disorders) consumes the C1 complement complex including C1q and C1-INH. In about two-thirds of AAE-C1-INH patients, anti-C1-INH antibodies (IgM, IgG, or IgA type) can be detected (30). The AAE-C1-INH due to anti-C1-INH antibodies was initially called autoimmune or type II AAE-C1-INH (31). Long-term follow-up of patients with AAE-C1-INH type II reveals that lymphoproliferative disease might develop later (21, 29). Therefore, these two types of AAE-C1-INH may overlap (31). The characteristic patterns of complement measurements are detailed in **Table 1**.

### Acquired Angioedema Related to Angiotensin-Converting Enzyme Inhibitors

AAE-ACEI is caused by elevated concentration of bradykinin, as the main enzyme responsible for its breakdown, the ACE, is inhibited. As no specific laboratory test is available for the identification of AAE-ACEI, this disorder can be diagnosed only by excluding other types of bradykinin-mediated angioedema (32, 33), when the complement tests are performed at the discontinuation of ACE inhibitors (**Table 1**) (32, 34). Furthermore, other vasoactive peptides degraded by ACE could be involved in AAE-ACEI (34).

### Idiopathic Non-Histaminergic Acquired Angioedema

The exact background and the molecular pathogenesis of AAE-InH are unknown. Similar to AAE-ACEI, the diagnosis of AAE-InH is based on excluding other disorders (**Table 1**), as well as on ascertaining that the edematous symptoms do not respond to the standard therapy with antihistamines and the family history is negative (35).

### Idiopathic Histaminergic Acquired Angioedema

AAE-IH is a mast cell and histamine-dependent, in which patients respond to short-term steroid, and it may be distinguishable from the angioedema associated with chronic spontaneous urticaria by the relative absence of IgG antibody to the IgE receptor or with IgG anti-IgE (35).

### Hereditary Angioedema With Normal C1-INH Function

The background of HAE-nlC1-INH is rather diverse as mutations of several genes (*F12, PLG, ANGPT1, KNG1, MYOF*, and *HS3ST6*) may lead to the different subtypes (32, 36). Therefore, complement testing should be performed in order to exclude C1-INH deficiency itself (**Table 1**), but there is no specific biochemical method for the exact diagnosis of HAE-nlC1-INH. Furthermore, the etiology of this disorder is unknown in a large percent of cases (32).

## Preanalytical Issues of Complement Laboratory Methods

The complex laboratory diagnosis of HAE-C1-INH requires a wide range of complement tests, which can be performed from different types of good-quality blood samples (37). Good quality means that after clotting (about 20–120 min), the serum must be separated by centrifugation as soon as possible and stored under controlled conditions.

The separated serum/plasma can be shipped (1) at room temperature for a maximum of 4 h or (2) frozen in case of longer transportation. Native serum properly prepared may be used for determining the concentration of C1q, C1-INH, C4, and anti-C1-INH antibodies, as well as for measuring the function of the complement pathways or the function of C1-INH. Besides the native serum, citrated plasma may be collected as well in order to analyze functional C1-INH levels: for this purpose, citrated blood should be centrifuged as soon as possible to separate platelet-free plasma. In case complement testing cannot be performed on the day of blood sampling, the serum and plasma samples must be stored in a deep freezer (−20°C) for up to 3 months or in an ultra-deep freezer (−70°C) for a longer storage until analysis. As functional tests are extremely sensitive, multiple freeze–thawing cycles should be avoided, by preparing several aliquots of each sample (37). In 2020, the utilization of the less invasive dried blood spot (DBS)-based assays has been introduced in the diagnosis of HAE-C1-INH, as a new study reported that enzyme activities can be retained in DBS samples as well (38). Its further advantage is that DBS samples may be transported and kept at ambient temperature without markedly affecting the sample’s quality.

## Available Biochemical Tests to Differentiate Subtypes

### Analyzing the Function of C1-INH

C1-INH function measurement is indispensable for a biological diagnostic of C1-INH deficiency. The distinction between type I and type II is achieved with a demonstration of the presence of an abnormal, non-functional protein (1, 2).

Based on their working principles, two types of commercially available tests are routinely used in the diagnostics and both work with the addition of surplus C1s to the citrate-anticoagulated plasma sample to be tested (39). These approaches are sensitive, as C1-INH is the exclusive inhibitor of C1r and C1s, in contrast with further serine proteases (kallikrein, factor XII, factor XI, MASP-1, MASP-2, plasmin, and thrombin) that are all regulated by C1-INH and other inhibitors. One of the methods detects the formed stable complexes between C1s and C1-INH, where avidin-labeled active C1s is added to the sample in surplus quantities. The avidin-labeled C1s enables the C1s–C1-INH complex to bind to the ELISA plate covered with streptavidin peroxidase, and finally, the bound complexes are detected with anti-C1-INH antibody. In case of the colorimetric method, the function of free (not bound to C1-INH) active C1s is monitored during a kinetic or endpoint assay, and a substrate is used that produces a color change (40, 41). The chromogenic assay is recommended for C1-INH function, while the discriminatory power between healthy and affected individuals using the complex ELISA may not be fully satisfactory for reliable C1-INH measurements (40, 41).

Further tests have been proposed for the determination of functional C1-INH concentration: as a first step, the functional C1-INH of the sample is pre-incubated with labeled kallikrein or with active factor XII. Thereafter, anti-C1-INH antibody is added to detect the C1-INH/enzyme complexes bound to the streptavidin-covered ELISA plate (42). Another test includes the purified form of contact-phase proteases and uses a synthetic substrate to measure the amidase activity not inhibited by C1-INH. In this method, the plasma samples are pre-incubated with a mix of protease inhibitors to block elevated kininogenase activity (43).

Most recently, a novel method has been introduced that enables measuring functional C1-INH activity in DBS samples: in detail, this approach analyzes the inhibitory activity on C1s by functional C1-INH present in the DBS sample using liquid chromatography-tandem mass spectrometry (LC-MS/MS) quantitation (38).

### Measuring C1-INH and C4 Concentrations

Serum concentrations of C1-INH or C4 are usually measured by using nephelometry/turbidimetry, radial immunodiffusion, and enzyme-linked immunosorbent assay (ELISA) using specific antibodies. When considering the differential diagnosis of angioedema subtypes, C1-INH and C4 concentrations should be considered always parallel with C1-INH function (1, 2, 44). Most recently, a novel and robust multiplexed assay was described that is capable to simultaneously analyze C1-INH, C1q, and C4 concentrations in DBS samples of HAE patients (38). In this approach, the blood proteins were extracted from tiny punches of DBS samples and were subsequently digested by trypsin. Finally, the signature peptide derived from C1-INH, C1q, or C4 is quantified by LC-MS/MS (45).

### Measurement of Anti-C1-INH Antibodies

ELISA plates covered with purified C1-INH are used for measuring anti-C1-INH antibodies, where the unbound antibodies from the patient sample are detected with different anti-human immunoglobulins, to show the presence of IgM-, IgG-, or IgA-type antibodies separately (46, 47). A few semiquantitative, ELISA-based methods have been introduced for analyzing the antibodies’ inhibitory effect on C1-INH (46, 48, 49), but these tests are not available commercially. Remarkably, the binding strength of the antibodies mostly determines the antibodies’ inactivating effect exerted on C1-INH, as well as the specific binding site on the C1-INH molecule (47, 50).

## Complement Determinations in Neonates and in Childhood

Diagnostic analysis of complement components and activities raises several issues considering the first year of life. Significantly lower classical pathway function (59% of the adult values) and decreased C4 concentration (64% of the adult values) were observed in full-term newborns, with even lower C4 in preterm neonates (40% of adult levels) (51). When considering the measurement of C1-INH from cord blood, its concentration was about 50%–60% of that observed in healthy subjects (52–54).

Furthermore, remarkable changes are also observed when determining the classical pathway’s function, as well as the C4 and C1-INH concentrations in subsequent cord blood samples of newborns and in samples of infants (55). Based on these data, we suggest performing repeated complement measurements with at least two consistent results before establishing the final diagnosis (the second test should be made after 1 year of age). These may be complemented with genetic analysis in those cases, where causative mutations could be identified in *SERPING1* in the family (55).

## Challenges Faced to Reach Diagnosis

Although an intensive effort has been done in the last years towards a rapid and precise diagnosis, misdiagnosis before being identified as having HAE-C1-INH has been reported (56). Allergic angioedema and appendicitis are the most frequent causes related to subcutaneous and submucosal edema, respectively (56). The delay on diagnosis is still a burden for HAE patients (57, 58). These findings suggest that additional investment must be done to improve the awareness of HAE.

Regarding laboratory diagnosis, complement is still the focus to select the affected patients instead of performing tests, which include kinin–bradykinin system. In both cases, sample collection and adequate manipulation represent a barrier for the diagnosis (55). Children could be truly identified as affected by HAE only after the first year of life if biochemical tests are used (50, 51). Genetics does not solve all the cases as well considering that sequencing could miss some mutations and not all HAE-nlC1-INH variants were identified, but it can be differential in some cases (32). For F12 variant carriers and during the pregnancy, observations of C1-INH decrease are not uncommon, mimicking a HAE-C1-INH situation.

## Perspectives of Tests for Bradykinin-Mediated Angioedema

A challenge step into the correct diagnosis of AE (especially in AE with normal C1-INH) is to distinguish if the swelling episodes are histamine- or bradykinin-mediated (59). Bradykinin is a vasodilator nonapeptide released from domain 4 of high-molecular-weight kininogen (HK) by plasma kallikrein hydrolytic activity. The estimated half-life of free bradykinin in plasma is shorter than 30 s (60, 61), which makes its measurement very challenging, hampering the determination of a bradykinin-mediated angioedema by the measurement of the peptide released in human plasma (62). In this context, the measurement of cleaved HK can be an alternative to identify an excessive release of bradykinin as the cause of HAE.

However, it is still not clear if the basal levels of free bradykinin/cleaved HK are high enough to distinguish between bradykinin-mediated and non-bradykinin-mediated HAE patients out of crises. Suffritti et al. (2014) analyzed by immunoblotting the profile of HK in the plasma of HAE-C1-INH patients and found a clear increase in the bands corresponding to cleaved HK (107- and 98-kD bands indicated by the authors) in samples collected during attacks (63). Although the authors report a percentual increase in the cleaved HK, HAE-C1-INH patient’s samples collected during remission showed a similar profile compared to the plasma of controls (major band around 130 kD and a faint band around 107 kD), jeopardizing the use of immunoblotting analysis for samples collected out of attacks (63). In contrast, another study where the HK cleavage was estimated by the abundance of cleaved HK species (corresponding to L chain), bands of 56-kDa and 45-kDa species were found in samples collected during attacks (64). They reported none or very low amounts of native HK in the plasma of HAE-C1-INH patients and observed a significant difference in the amounts of native and cleaved HK between controls and HAE-nlC1-INH samples, with an additional significant difference between men and women (64). Importantly, immunoblotting technique for quantitative analysis requires many quality controls checks such as validation of the integrity of the sample, the specificity of antibodies, linearity of sample loading, and densitometry analysis (65, 66). Although both mentioned studies (63, 64) collected the blood samples in citrate tubes, there is a notable difference in the manipulation of samples regarding the time between collection and centrifugation of the plasma. In the Baroso et al. (64) study, the citrated blood samples were shipped at room temperature (20–25°C) within 2 days before being centrifuged, whereas Suffritti et al. (63) collected the blood in citrate tubes containing a protease inhibitor cocktail and centrifuged the samples within 1 h. Therefore, the diagnosis of bradykinin-mediated angioedema based on the use of HK immunoblotting still lacks a consensual standardization.

An interesting alternative for the measurement of cleaved HK is the use of LC-MS to specifically detect the 46-kD fragment corresponding to the HK low chain (final product of plasma kallikrein hydrolysis) (64). The measurement of cleaved HK by LC-MS is an interesting strategy, since it eliminates the variables related to the variation of the antibodies used in immunoblotting, as well as sample loading and quantitation variables. Preliminary results showed a potential capacity of LC-MS to distinguish between controls and HAE-C1-INH plasma by measuring the 46-kD low chain of cleaved HK (64).

Another proposed approach to address bradykinin-mediated angioedema is the analysis of spontaneous amidase activity in plasma. The measurement of spontaneous amidase activity refers to the ability of different serine proteases, such as plasma kallikrein, factor XII, plasmin, and tissue plasminogen activator to hydrolyze specific synthetic peptides *in vitro* (usually HD-Pro-Phe-Arg-*p*NA or Z-Phe-Arg-AMC-HCl), and it is frequently used to evaluate the activation of the kallikrein–kinin system (65–67). Since most of the serine proteases circulate as zymogens in plasma, the activation of the proenzymes of the kallikrein–kinin system can be achieved by the addition of negatively charged molecules such as dextran sulfate. Joseph et al. (2013) demonstrated a spontaneous production of plasma kallikrein in virtually all HAE-C1-INH patients as well as in diluted normal plasma, in a stoichiometric mechanism of prekallikrein activation independent of factor XII (68).

In another study, a significant increased spontaneous amidase activity was also observed in the citrated plasma of patients with HAE-C1-INH and AAE-C1-INH compared to controls. Although significantly higher when compared to the controls, the spontaneous amidase activity of HAE-nlC1-INH patients was quite lower compared to HAE-C1-INH (69). In addition, it was reported that the use of oral contraceptives containing estrogen may increase the spontaneous amidase activity for some HAE-nlC1-INH patients (69).

Another study subsequently published showed a similar response for HAE-C1-INH during remission and attacks (63), but the difference found was not enough to establish a normal range and a threshold for a normal/high spontaneous amidase activity. In this study, the measurement of the plasma capacity to inhibit exogenous plasma kallikrein showed a better capacity to distinguish between controls and HAE-C1-INH patient’s samples during remission and attacks (63). Another approach involving the activation of the kallikrein–kinin system proposes that a specific dose of 2.5 μg/ml dextran sulfate is enough to stimulate a maximal amidase activity able to discriminate the plasma from bradykinin-mediated angioedema patients from controls and non-bradykinin mediated, while lower doses were able to stimulate only HAE-C1-INH plasmas (70). The stimulated amidase activity efficiently distinguished samples from HAE-C1-INH, HAE-nlC1-INH, and AAE-InH patients from controls and histaminergic patients, whereas the spontaneous amidase activity was only significantly higher in the HAE-C1-INH group (63). Noteworthy, the plasma samples used in the dextran sulfate-stimulated amidase activity were collected in EDTA tubes and centrifuged and frozen within 15 min (70).

A commercial test based on the spontaneous amidase activity and the proenzyme activatability (69) reports a sensitivity of 80%–81% and specificity of 91%–100% for general bradykinin-mediated angioedema and a sensitivity of 74%–75% and a specificity of 91%–99% for angioedema with normal C1-INH (Kininogenase kit, KininX SAS).

The degradation profile of the serum glycoprotein 120 (sgp120) by incubation with plasma showed a linear correlation with the spontaneous amidase activity in samples of HAE-C1-INH patients (71). When incubated at 4°C in plastic tubes, HAE-C1-INH plasma was clearly able to cleave sgp120, while control plasmas did not cleave sgp120 after 12 h of incubation. However, not all the HAE-nlC1-INH plasma samples were able to cleave the sgp120 at the same conditions, including HAE-F12 and HAE-PLG samples (71).

## Take Home Messages

- Biochemical assays evaluating complement activation are the recommended tests for HAE diagnosis yet.
- Manipulation of the samples represents a critical step for HAE diagnosis.
- Genetic variants are not identified in every patient, but may be differential in HAE-nlC1-INH diagnosis.
- Kinin–bradykinin assays could improve the knowledge of pathomechanisms involved in HAE.

## Author Contributions

AG, CV, and HF contributed to the conception and design. CV, DC, AG, and HF developed the first draft. AG, CV, DC, and HF worked together on the final version of the manuscript. All authors contributed to the article and approved the submitted version.

## Funding

HF was supported by the National Research Development and Innovation Office (NRDIO) Grant No: K124557.

## Conflict of Interest

The authors declare that the research was conducted in the absence of any commercial or financial relationships that could be construed as a potential conflict of interest.

The handling editor declared a past co-authorship with the author AG.

## Publisher’s Note

All claims expressed in this article are solely those of the authors and do not necessarily represent those of their affiliated organizations, or those of the publisher, the editors and the reviewers. Any product that may be evaluated in this article, or claim that may be made by its manufacturer, is not guaranteed or endorsed by the publisher.

## Abbreviations

AAE-ACEI, angioedema induced by angiotensin-converting enzymes (ACE) inhibitors; AE, angioedema without urticaria; AAE-C1-INH, acquired angioedema with C1-INH deficiency; AAE-IH, idiopathic histaminergic acquired angioedema; AAE-InH, idiopathic non-histaminergic acquired angioedema; C1-INH, C1 inhibitor; HAE, hereditary angioedema; HAE-C1-INH, HAE with C1-INH deficiency; HAE-F12, HAE with *F12* gene mutation; HAE-nlC1-INH, HAE with normal C1-INH; HAE-PLG, HAE with *PLG* gene mutation; HK, high molecular weight kininogen; KK, plasma kallikrein.
